# Odontological Society of London

**Published:** 1857-07

**Authors:** 


### ARTICLE IX.

### *Odontological Society of London.*

*Monday, April* 6, 1857.

Mr. Arnold Rogers in the chair.

In the absence of the author, (Mr. Maclean,) Dr. Barnett read the paper "On the systematic removal of the four permanent first molars at an early period, in overcrowded arches, especially when attacked by caries." The author commenced by stating that he was far from advocating any officious interference with nature, that so long as the teeth in question, though affected by caries, appeared capable of being saved, or the arch, though limited in extent, seemed likely in time to develop itself sufficiently to accommodate the full number of teeth, so long he advocated non-interference, as from practical observation he had full confidence in the power of nature to place the teeth in their normal position in arches of sufficient capacity. He next proceeded to detail the advantages connected with the practice he advocated. These advantages are as follows:

1. The prevention and connection of the simpler forms of irregularities in the easiest and most desirable way, in a great majority of cases without the aid of mechanical means, in all in such a manner, as least to disfigure the appearance of the mouth.

2. The promotion of a healthier state amongst the remaining teeth, and an increase in the facility of treating caries when it presents itself.

3. The prevention of the distressing, and in some cases even very serious symptoms which frequently accompany the development of the wisdom teeth in overcrowded arches, and a material diminution in the chances of the formation of sinuses in after life.

The statements of the author were supported and illustrated by casts taken before and after the operation in question had been performed, and exhibiting its beneficial results, and also by others showing the effects of a different line of treatment, in which sound anterior teeth had been extracted for the cure of irregularity, and diseased first molars left.

Mr. Tomes said the tables referred to by the author were calculated from hospital cases, and in practice of that kind the operator was not at liberty to extract teeth for the mere cure of irregularity. The working classes sent their children to the hospital when suffering from tooth-ache, and he (Mr. Tomes) would not, under these circumstances, venture upon the removal of the first molars in all children who had crowded arches.

Mr. E. Saunders said it could not be denied that the first molar was very liable to fail at an early age, but he was not clear that the causes of its early failure had been sufficiently made out. If it was said that the tooth was formed at a period of life unequal to the production of sound bone, it might be expected that the central incisors would fail as frequently; which could hardly be asserted. He believed the main cause was, inattention to the first set of teeth—the neglect of those sanitary measures, the necessity of which was universally recognized in regard to the second set. When asked if children should brush their teeth, his reply was, should their faces be washed? The habitual neglect of the first set of teeth, even amongst persons of education and refinement, constantly led to the decay of the temporary molars, thus producing an early failure of the permanent molars, which in their early stage had a morbific *entourage* acting upon them, preventing their healthy development. He could not give in his adhesion to the propriety of removing the first permanent molar, as he thought it was always accompanied by a prejudicial narrowing of the arch. The removal of one of the two bicuspids, which so frequently failed, would give all the room that could be desired, and leave the arch more efficiently maintained.

The Chairman said it was notorious that the six-year-old molar tooth, amongst all classes, was almost always defective; yet scarcely two persons were agreed as to the cause of the defect.

Mr. Rogers, jr. said he observed from the casts exhibited, that after the removal of the six-year-old molars in the lower jaw, there was a greater tendency on the part of the twelve-year-old molars to fall forwards. The operation seemed to succeed better in the upper jaw than the lower, as regards the subsequent shape of the mouth.

Mr. Harrison considered that in the matter under discussion, the principle *in medio tutissimus ibis* would be found correct. He should not be disposed to condemn the first molars, as a general rule, on the supposition that the bicuspids were more durable teeth. If, however, they were decayed, no one would hesitate to extract them. If both the molars and the bicuspids were perfect, he should rather sacrifice one of the latter, which would ordinarily give all the room required, and less pain would be occasioned to the patient. It was certain that the permanent molar of the present day was much more liable to run into premature decay than formerly. The operator should be guided by the actual circumstances of each case, and no general rule should be laid down applicable to all.

Mr. Duff thought that unless we could arrive at some satisfactory reason for supposing the permanent bicuspid much more durable in its structure than the permanent molar, the dentist would not be justified in removing the latter in preference to the former. The posterior edge of the permanent molar frequently impinged upon the anterior edge of the second bicuspid, causing a derangement of both, in which case he thought the removal of the second bicuspid would be the best mode of practice. He had lately seen a lamentable case in which a medical man had allowed a medical friend, not a dentist, to pull out the permanent molars of his daughter, who was about thirteen years old. She had a nice set of teeth, with no overcrowding of the arch; yet she had not a single molar left, and they would never come again. For himself, he should be guided by the form of the mouth. If the bicuspid was more out of place than the molar, he should remove it; but not otherwise.

Mr. Tomes said that Dr. Ashburner, in his work on diseases consequent on dentition, was the great advocate of the removal of the first molar, whether decayed or not, in all cases where children were affected with glandular swellings about the neck. He had seen some cases so treated in which the second molars had come forward into the vacated place, and the first did not appear to be very much missed; yet he was not prepared to advocate the practice. The tables that had been referred to were taken from 3000 cases. He had collected, in all, twelve or fourteen thousand, but had not had time to classify them; he believed, however, that the same general result would be arrived at. Mr. Underwood, in a paper in the "American Journal," had published similar tables with pretty much the same results as those published by him (Mr. Tomes.)

Mr. Woodhouse did not agree with the author as to the time of the removal of the first molars. Children were often brought to the dentist, for pain and tenderness in the teeth, long before the temporary molars were being changed; and his practice was, to prepare these teeth slightly, by stopping them with cement, so that they might last until the bicuspids became efficient as molars; and then to remove them. In this way, children were able to masticate properly; which was of great importance at their time of life; and, when the teeth were removed, they were stronger and better able to bear the operation.

Mr. Samuel Cartwright, Jr., said, the propriety of removing the molar or bicuspid tooth depended upon the state of the mouth, and it was impossible to lay down general rules. The canine tooth had something to do with it. If the bicuspid lay close to it, it was the tooth, he thought, which should be sacrificed.

Mr. Barrett said, it had not been stated how often the molar tooth decayed after attempts had been made to preserve it. At the age when, the author stated, it was necessary to remove these teeth, there were no others to supply their place, the second molar not having made its appearance; so that, by the practice recommended, the masticatory power of the mouth was destroyed, the onus of mastication being thrown upon the front teeth, which, if irregular, would not, to say the least, be improved by the process. There was another objection to the practice, which might be mentioned—namely, that the wisdom teeth were also extremely liable to decay; so that, if both were lost, four large and able teeth were sacrificed in order to bring into operation four smaller and often fragile teeth.

Mr. Saunders said the durability of the wisdom tooth depended very much upon the crowded state of the mouth. If it was very far back, and imperfectly attended to, it was very liable to decay; but if the first molar was removed early, so that the wisdom tooth came more forward in the mouth, it was not so liable to fail. Where the first molar was affected with lateral decay, and the posterior surface of the second bicuspid was implicated in the same mischief, the best plan was to remove the second bicuspid, leaving free space, and giving a chance of success in the efforts to save the molar.

Mr. Martin said that when the anterior molar was removed, the wisdom tooth developed itself more freely, and there was frequently a much better and more valuable formation. In many cases, the loss of the wisdom tooth was owing to the fact that it was not able to develop itself, from the peculiarity of its position and the comparative difficulty of removing portions of decomposed food from its surface.

Mr. Bigg concurred with the observations of Mr. Martin; but suggested that they did not bear upon the case mentioned by the author. It had been frequently observed that decay existed in the first molars very shortly after formation, and it was extremely difficult to arrest its progress. He had tried cement for a time; but it had often produced irritation, and he had been compelled to extract the teeth afterwards. In certain conditions of the mouth, the first molar should be the tooth removed; but there were probably other cases in which, from the formation of the mouth, the first or second bicuspid should be removed instead. Each case should be left to the judgment of the practitioner. He had seen cases in which great mischief had been occasioned by the premature removal of the first molar. Dentists should be exceedingly careful, before operating, to see which teeth were faulty. He had lately seen a boy, fourteen years old, who was taken to a dentist, who extracted the first bicuspid, which was found to be perfectly sound. He had neglected to examine the first molar, which was exceedingly carious. He found the case very difficult to treat, and he should like to ask the opinion of the members as to what should be done when the teeth, shortly after having been stopped, began to decay again in some other place.

The discussion on the paper was adjourned to the next meeting, the Chairman promising to give the result of his experience on the subject.

Mr. Barrett read a paper "on the advantages of being able readily to adjust the head of the patient to any position required by the operator, and also on the means for effecting this." The advantage of having an operating-chair so constructed as readily to adjust the patient to any required position is shown by the difficulty daily experienced when we have to operate upon persons either much above or much below the average height, supposing the parts of the chair to be fixed. The first point in importance, therefore, is to be able to place the head of the patient, whether he be six feet high or only three feet, upon the same level. Unless this can be done, the operator must make his own height conform to that of the patient. The continued performance of operations in the constrained and unnatural positions thus required not only lessens the capability for performing lengthened and delicate operations, but must tend to affect seriously the health of the operator. The position in which the patient should be placed is necessarily governed by several circumstances, viz. the height of the operator, the height of the patient, the direction of the light, &c. The chair exhibited to the society this evening is so constructed as to be made readily conformable to these conditions; for this purpose its movements are as follow:

| | |
|---|---|
| The seat will move | upwards—downwards. |
| The back " | upwards—forwards.<br>downwards—backwards. |
| The head-piece | forwards—backwards. |

The seat can be raised or lowered nine inches, commencing from the ordinary level, by the pressure of the foot upon a pedal at the base of the chair, the motion being performed with little exertion on the part of the operator, and almost imperceptibly to the patient whilst sitting in the chair. Thus, instead of stooping to operate upon the upper teeth, we raise the patient to a height which will allow the operator either to sit or stand; instead of standing upon tiptoe or a footstool to reach the lower teeth, the patient himself is lowered to the most convenient level. The upward back movement is seldom required, but is found advantageous when operating upon the upper teeth of the tall patient, as the head can then be supported at a higher level than could otherwise be obtained; it also serves to adjust the curve of the chair-back to the spine of the patient. A few revolutions of a handle placed behind the chair will suffice to raise the back five inches. The forward or backward movement enables us to place the patient at any required angle of inclination—a matter of great importance when the light comes into the room in an almost vertical direction. The head-piece movement is most frequently called into requisition, and by its peculiar construction serves to carry out with extreme facility, but in a small degree, all those adjustments effected by the parts already described. The movement is obtained by detaching, with the pressure of the thumb, a pin, which secures the head-piece by entering a perforated segment plate. The head-piece can then be rotated freely, but upon withdrawing the pressure of the thumb from the pin the head-piece again becomes fixed in any position required. The centre of the shaft, upon which the head-piece rests, being curved from the axis upon which it revolves, an eccentric motion is produced, by which an additional height of three inches can be instantly obtained, without raising the back of the chair or lowering the seat. The head of the patient, by means of this head-piece, can be placed in a position almost horizontal with the body, enabling the operator to apply his force with the greatest freedom either for removing or stopping the upper teeth; at the same time the eye is enabled to follow unobstructedly the movements of the instrument. This opportunity seemed favorable to introduce to the society the name of Mr. Betjemann, of 28 Oxford street, to whose persevering industry the author is indebted for the chair exhibited this evening. It is believed that all will justly estimate the services of that skillful artizan, who, possessing an unusual amount of mechanical ingenuity, has also acquired a great facility for embodying any ideas which may be given to him. The author feels confident that these conditions will be realised whenever the services of Mr. Betjemann may be required.

In answer to questions from several members,

Mr. Betjemann, the maker of the chair, stated that its cost would be about thirty guineas, and gave some further explanations as to its machinery and movements.

Mr. Tomes said he had a chair, constructed at a cost of fifty guineas, which fulfilled many of the conditions of Mr. Betjemann's chair, the whole of the movements being effected by screws. But in several respects it was not perhaps quite so convenient as the one exhibited to the society.

Mr. Coleman suggested a method in which the movements would be effected by a series of air-cushions, inflated by a reservoir, in the form of a gasometer, to be kept in an adjoining room.

Mr. Saunders stated that his chair had an ascending back, made on the principle of an artist's easel, the back sliding within an outer frame, assisted by a spring rack, having a range of some nine or ten inches.

The following gentlemen were elected members of the society: Stratton Coles, Esq., (Plymouth;) William Crampton, Esq., Grosvenor street; W. H. Scott, Esq., 23 Wimpole street; G. McAdam, Esq., (Hereford;) James Orrock, Esq., (Nottingham;) Fred. Oswin, Esq., 27 Harley street; J. Barker, Esq., New Bridge street. The society then adjourned.

The next meeting will take place Monday, May 4th.—*Brit. Jour. Dent. Sci.*

